# Better long-term survival in young and middle-aged women than in men after a first myocardial infarction between 1985 and 2006. an analysis of 8630 patients in the Northern Sweden MONICA Study

**DOI:** 10.1186/1471-2261-11-1

**Published:** 2011-01-05

**Authors:** Rose-Marie Isaksson, Jan-Håkan Jansson, Dan Lundblad, Ulf Näslund, Karin Zingmark, Mats Eliasson

**Affiliations:** 1 The Northern Sweden MONICA Myocardial Registry, Department of Research, Norrbotten County Council, Luleå, Sweden; 2 Department of Nursing, Umeå University, Umeå, Sweden; 3 Department of Medicine and Geriatrics, Skellefteå Hospital, Skellefteå, Sweden; 4 Department of Medicine, Sunderby Hospital, Luleå, Sweden; 5 Department of Cardiology, Heart Centre, University Hospital, Umeå, Sweden; 6 Department of Public Health and Clinical Medicine, Umeå University, Umeå, Sweden; 7 Department of Research, Norrbotten County Council, Luleå, Sweden

## Abstract

**Background:**

There is conflicting and only scant evidence on the effect of gender on long-term survival after a myocardial infarction (MI). Our aim was to analyse sex-specific survival of patients for up to 23 years after a first MI in northern Sweden and to describe time trends.

**Methods:**

The Northern Sweden MONICA Myocardial Infarction Registry was linked to The Swedish National Cause of Death Registry for a total of 8630 patients, 25 to 64 years of age, 6762 men and 1868 women, with a first MI during 1985-2006. Also deaths before admission to hospital were included. Follow-up ended on August 30, 2008.

**Results:**

Median follow-up was 7.1 years, maximum 23 years and the study included 70 072 patient-years. During the follow-up 45.3% of the men and 43.7% of the women had died. Median survival for men was 187 months (95% confidence interval (CI) 179-194) and for women 200 months (95% CI 186-214). The hazard ratio (HR) for all cause mortality after adjustment for age group was 1.092 (1.010-1.18, *P *= 0.025) for females compared to males, *i.e*. 9 percent higher survival in women. After excluding subjects who died before reaching hospital HR declined to 1.017 (95%CI 0.93-1.11, *P *= 0.7). For any duration of follow-up a higher proportion of women were alive, irrespective of age group. The 5-year survivals were 75.3% and 77.5%, in younger (<57 years) men and women and were 65.5% and 66.3% in older (57-64 years) men and women, respectively. For each of four successive cohorts survival improved. Survival time was longer for women than for men in all age groups.

**Conclusions:**

Age-adjusted survival was higher among women than men after a first MI and has improved markedly and equally in both men and women over a 23-year period. This difference was due to lower risk for women to die before reaching hospital.

## Background

Mortality from myocardial infarction (MI) in Sweden, as in most other developed countries, has decreased markedly during recent decades[1]. The international MONICA collaboration hypothesizes that the decline is mainly due to a decrease in the classical risk factors -- smoking, cholesterol and blood pressure [2], although recent modelling strongly emphasizes the pivotal role of lower cholesterol [3]. However, MI still remains the most common cause of death in Sweden and other western regions [4]. Women are affected by MI later in life than men. Below 55 years of age the risk of MI in men is almost four times that of women [5]. With increasing age, the incidence of MI increases markedly, but the incidence and mortality from MI remain lower in women throughout life [5].

Most studies of long-term survival after an MI have had a follow-up time of maximum 5 years [6-10], only a few have had a longer period [11-14]. Furthermore, most have included both the first and recurrent MIs [6,10,11,13,14]. Only few studies have documented the impact of sex in long-term survival after an MI [6,10,11,15]. Two of these studies from the 1990 s [6,10] included first and recurrent MIs, had a short perspective and found no sex differences in the 30-day to 1-year mortalities. A Scottish study of 201 114 patients with a first MI during the years 1986-1995, covering also initial out-of-hospital deaths, showed that women did not fare worse than men when age was taken into account [15]. In an American study including 12 331 patients with both first and recurrent MIs, with a 6-year follow-up, long-time survival was also similar between the sexes [11].

No studies have investigated sex-specific time trends in long-term mortality in patients with a first MI. As there are important sex differences in mortality before admission to hospital, men fare worse [15], such data must be included to give the true picture of the total mortality burden of ischemic heart disease (IHD). To our knowledge only one study has taken a specific sex and age approach when analysing trends in long-term survival after a first MI [12]. This recently presented Norwegian hospital-based study included 12 000 patients, divided into two age groups (<60 years, >60 years) with a first MI. However, pre-hospital mortality was not included. A substantial decline in long-term mortality in both sex and age groups over 23 years was found but mortality did not differ between men and women.

The aim of this study was to analyse sex-specific long-term survival up to 23 years in patients below 65 years of age with a first MI during 1985-2006 in northern Sweden. In addition, we aimed to study if trends over time differed between sexes.

## Methods

This study relies on two separate registries in Sweden: The Northern Sweden MONICA Myocardial Infarction Registry and The Swedish National Cause of Death Registry.

The WHO MONICA project (Monitoring Trends and Determinants in Cardiovascular Disease) started in the beginning of the 1980 s. The Northern Sweden MONICA Study is ongoing in Västerbotten and Norrbotten counties since 1985. The Myocardial Infarction Registry includes cases based on the MONICA criteria [16].

The registry is population based, which means that all MI events are included, not only those who are admitted to hospital. Most importantly, all deaths out of hospital are evaluated for possible myocardial infarction. The case findings of possible events are based mainly on two sources, hospital discharge registries and death certificates. To validate the diagnosis of MI, medical history, clinical symptoms, ECG and cardiac enzymes are used based on strict WHO MONICA criteria throughout the period. MI diagnoses are based on typical symptoms and biomarkers for myocardial necrosis. If only one of these parameters is positive, ECG analysis is included for final diagnosis.

In fatal cases, information obtained from death certificates and necropsy reports, when available, is also included. In fatal events, possible infarction and unclassifiable infarctions are included, according to the international MONICA protocol. An event is considered first ever if the history according to the hospital records and the MONICA database is free from previous clinically recognized MI, otherwise the event is considered as recurrent [16]. Since 2000 all the reporting hospitals have switched to the use of troponins, and the local reference values are applied.

During the study period from January 1, 1985 to December 31, 2006, a total of 12 635 patients who fulfilled the MONICA criteria and diagnosed as a definite MI were registered in the MONICA myocardial infarction registry. In this study only first ever MIs in subjects 25-64 years of age were included. Hence, a total of 8630 patients were included. The population at-risk in the area was 258 576 (December 31, 2009). Data on clinical characteristics were not routinely registered before 1989.

All patients were followed up for information on vital status using The Swedish Cause of Death Registry until August 30, 2008. Patients not identified in the register were assumed to be alive at that date and censored. It is estimated that 93% of all deaths are reported to Statistics Sweden within 10 days and 100% within 30 days using the unique personal identification number (PIN) assigned to every citizen in Sweden [17]. The two registries (MONICA and The Cause of Death Registry) were linked by the PIN. The endpoint studied was death from any cause, *i.e*. all cause mortality.

### Ethical considerations

The Northern Sweden MONICA Study was approved by the Research Ethics Committee of Umeå University, and the data handling procedures were approved by the National Computer Data Inspection Board. Participants or relatives to nonsurvivors gave written consent.

### Statistical analyses

Separate groups according to age at onset were constructed to achieve groups of similar size: 25-50 years, 51-56 years, 57-60 years and 61-64 years for men and women. The patients were separated into four cohorts representing the year of onset of MI: 1985-1988, 1989-1994, 1995-2000 and 2001-2006. Survival time was calculated as the number of days between the date of the MI and the date of death, or August 30, 2008.

Survival times were computed with Kaplan-Meier product limit estimate. Hypothesis test of no difference in survival was calculated by Cox regression analysis, with adjustment for age group. For explanatory reasons we performed a sensitive analysis by omitting those who died out of hospital and repeated the survival analysis. All analyses were carried out using the statistical computer program PASWStatistics 18.

## Results

A total of 8630 subjects, 6762 men and 1868 women, with a first MI between 1985 and 2006, were included. Mean age for men was 55.5 years and for women 56.4 years. Clinical characteristics are shown in Table 1. No data, except age, were available for the first period, and missing data ranged from 1 to 16%. Age at onset was stable for men but declined slightly among women over time.

**Table 1:** Clinical characteristics at baseline of men and women with a first myocardial infarction during four consecutive time periods, 1985 to 2006*

		1985-1988	1989-1994	1995-2000	2001-2006	1985-2006
**Men**	*N*	1463	1927	1711	1662	6763
	Survived (%)	408 (27.8)	903 (46.9)	1092 (63.8)	1296 (78.0)	3697 (54.7)
	Age (%)	55.60	55.30	55.30	55.70	55.50
	IHD (%)		355 (30.1)	520 (31.3)	412 (25.3)	1287 (28.8)
	Hypertension (%)		782 (42.6)	531 (33.4)	598 (36.9)	1911 (37.9)
	Diabetes (%)		221 (11.6)	242 (14.2)	262 (15.9)	725 (13.8)
	Regular smoking (%)		683 (38.2)	545 (43.0)	516 (38.2)	1744 (39.6)
**Women**	*N*	350	509	495	514	1868
	Survived (%)	101 (28.9)	230 (45.2)	338 (68.3)	401 (78.0)	1070 (57.3)
	Age	57.10	56.50	55.90	56.20	56.40
	IHD (%)		86 (27.5)	138 (29.0)	127 (25.2)	351 (27.2)
	Hypertension (%)		247 (50.9)	196 (42.4)	231 (47.2)	674 (46.9)
	Diabetes (%)		82 (16.3)	71 (14.4)	97 (18.9)	250 (16.6)

* Missing data: IHD 15.5%, hypertension 5%, diabetes 0.6%, smoker 16%.

Previous known ischaemic heart disease (*i.e*. angina pectoris) was initially more common among men and then similar between the sexes. The prevalence of hypertension declined but was more common among women during the whole study period. Known diabetes was more common among women and increased somewhat over time. Regular smoking was much more common and increased among women, while it was stable in men. No data were available on the prevalence of hyperlipidaemia or treatment with lipid-lowering drugs.

Median follow-up was 85 months (7.1 years) and maximum 288 months (24 years). Total follow-up was 70 002 patient-years. During the follow-up a total of 3066 (45.3%) of the men and 798 (42.7%) of the women died. Among men 959 (14.2%) died before admission to hospital and among women 205 (11.0%). Mean age for those who died before reaching hospital was 56.0 years for men and 57.1 years for women.

### Survival according to sex

In a Cox regression analysis the hazard ratio (HR) for all-cause mortality, after adjustment for age group, was 1.09 (95%CI 1.010-1.18, *P *= 0.025) for females vs. males, *i.e*. 9 percent higher survival in women than in men. An analysis using age and year of onset as time-dependent variables gave the same results (data not shown). After excluding subjects who died before reaching hospital HR declined to 1.017 (95%CI 0.93-1.11, *P *= 0.7).

**Figure 1:**
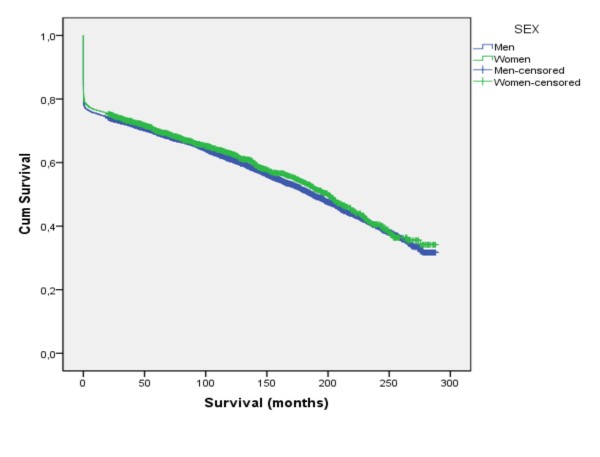
**Median survival after a first myocardial infarction according to sex**.

Median survival for men was 187 months (95% CI 179-194) and for women 200 months (CI 186-214) (Figure 1). In Table 2 the proportions that were alive at 7 days, 28 days, 1, 3, 5, 10 and 20 years after their MI are presented, stratified for age. For any duration of follow-up a higher proportion of women were alive, irrespective of age group. As the median follow up was 7.1 years figures on 5-year survival will include most subjects. Five years after a first MI 75.3% and 77.5%, among younger (<57 years) men and women were alive, and 65.5% and 66.3% in older (57-64 years) men and women were alive, respectively. After excluding subjects who died before reaching hospital median survival for men was 225 months (95% CI 217-233) and for women 222 months (95% CI 207-236).

**Table 2:** Proportion of men and women surviving after a first myocardial infarction stratified for age

	Survival (%) at different times after MI			
	
	Men		Women	
		
Time after MI	Age 25-55 years	Age 56-64 years	Age 25-55 years	Age 56-64 years
7 days	82.2	77.6	85.2	79.1

28 days	81.2	74.9	82.9	76.9

1 years	79.2	72.3	81.4	73.5

3 years	77.3	68.9	79.3	69.9

5 years	75.3	65.5	77.5	66.3

10 years	71.0	58.0	75.1	59.8

20 years	63.9	48.9	69.7	51.2

### Survival according to age group

**Figure 2:**
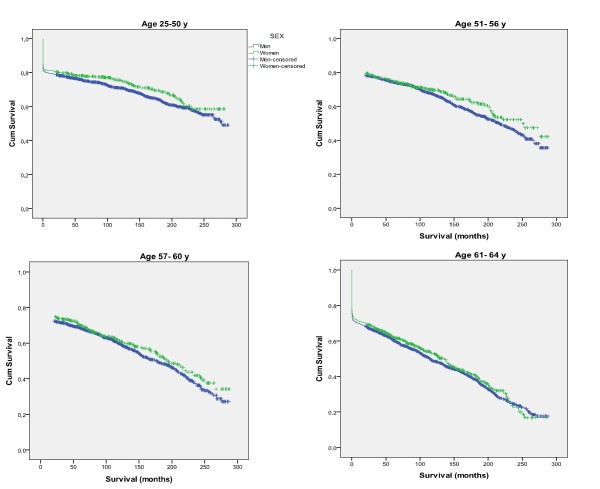
**Survival after a first myocardial infarction according to sex and age group**.

In each age group women had a longer median survival than men (Table 3, Figure 2). Survival was shorter with higher age.

**Table 3:** Median survival in men and women after a first MI according to age group

Survival (months)		
**Age at onset**	**Sex**	**Median**

25-50	Men	278
	
	Women	NA
	
		

51-56	Men	217
	
	Women	250
	
		

57-60	Men	177
	
	Women	192
	
		

61-64	Men	115
	
	Women	136
	
		

25-64	Men	187
	
	Women	200

### Survival according to cohort

**Figure 3:**
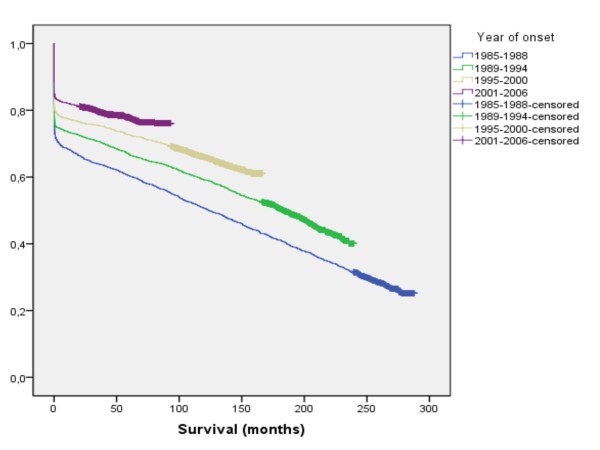
**Survival after a first myocardial infarction according to year of onset**.

**Figure 4:**
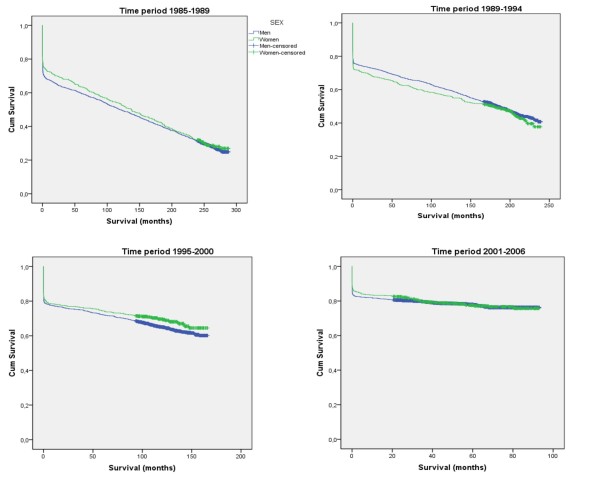
**Survival after a first myocardial infarction according to sex and year of onset**.

For each cohort survival increased (Figure 3). During the first and third periods survival in women exceeded that of men, while the reverse was true for the second period, and no difference was seen during the last period (Table 1, Figure 4).

## Discussion

Between 1985 and 2006 long-term survival after a first MI has increased in both men and women between 25 and 64 years of age in northern Sweden. Over the whole 23-year period women showed a nine percent higher survival than men. This difference was due to lower risk for women to die before reaching hospital, and during the last period similar rates of long time survival were noted in men and women.

During the last two decades differences in outcome between men and women suffering an MI have been increasingly highlighted. Still the evidence is conflicting regarding gender specific outcomes and prognosis after MI. Comparison between studies is complicated by methodological differences such as differences in data collection, registration methods, study population, case definition and research period. However, no study has previously presented data from such a large database as the Northern Sweden MONICA Study, over such a long time as 23 years, including exclusively patients with their first MI, and including also patients who died before reaching the hospital.

Improvement in long-term survival after a first MI is probably attributable to a combination of better treatment before admission to hospital by paramedics, improvements in the acute coronary care, and secondary prevention. Over the study period there was an increasing use of evidence-based medical treatments and coronary artery revascularisation at the onset of MI, all of which have been shown to reduce cardiac morbidity and mortality [18]. Secondary prevention programmes and patient activities have become routine in the follow-up of MI patients in Sweden. Since the early nineties all patients have been called and invited for individual counselling by cardiologists or dedicated nurses, for educational programs and for exercise programs in groups. Prescriptions for platelet inhibitors, beta-blockers, ACE-inhibitors and statins have increased in parallel with evidence and publications of landmark studies [3]. The attendance rate at clinical follow-up after an MI in Sweden has been generally high, approximately 80-90%, although patients with lower socioeconomic status have had a lower participation rate.

The increased long-term survival as shown in our study also reflects the forceful implementation of primary and secondary prevention of MI which has been shown in a recently published modelling study from Sweden [3]. Approximately 36% of the decrease in mortality in coronary heart disease during 1986-2002 was due to treatment of individuals and 55% was due to population risk factor reductions including total cholesterol, smoking and blood pressure levels. The substantial reduction in total cholesterol level explained almost 40% of the decrease, and almost 10% of the mortality reduction came from a decline in smoking prevalence.

Interestingly, during the studied 23-year period, the incidence of first MI before the age of 65 years in northern Sweden decreased among men but was unchanged among women [16]. In the Northern Sweden MONICA study we recently presented data on time trends in major cardiovascular risk factors from six population surveys covering the same population and time period as the MI registry. Significant improvements were observed in the control of hypertension, smoking, and an increased level of education between 1986 and 2009 noted [19]. In spite of increasing BMI no increase in the prevalence of diabetes was found. Total cholesterol levels decreased 0.9 mmol/l in both men and women, which is likely to have a major impact on the improved prognosis over time. Lower risk factor levels were achieved in both men and women although the decline in smoking was much slower in women. In 2009 more women than men were still regular smokers. This may be a factor behind our finding of no difference between men and women during our last observation period.

During the last two decades there has been an ongoing debate about the differences between men and women suffering from MI in terms of treatment and outcome [20,21]. It has been repeatedly stated that women with MI are treated less aggressively than men and that reperfusion therapy is under-utilized in women with MI [22]. Women are also said to be less likely to be referred for coronary angiography and revascularization procedures such as percutaneous coronary interventions and coronary artery bypass grafting [23]. However, one recent Swedish study with 1744 patients reported that men and women were treated similarly [24].

Our results do not support the contention that the outcome of a first MI in women is worse than in men. On the contrary, we found a slightly longer median survival and higher probability of being alive during long-term follow-up in accordance with recent large studies [11,12,15]. Excluding subjects dead before arrival to hospital abolished all gender differences but women still did not fare any worse than men. This was expected, as it was previously known that men had a higher proportion of out-of-hospital deaths. This has important implications for interpretation of studies based solely on those admitted to hospital in which an apparent worse prognosis for women has been evident. It also underscores the unique properties of the Northern Sweden MONICA registry mirroring the total mortality burden of a first MI in the society.

The reasons for the difference in long-term outcome between men and women are unclear but higher fatality in men before reaching of hospital surely contributes. Further explanations to a great extent are speculative. We had no data on severity of the MI which may be a link in the causal chain leading to somewhat better prognosis for women. Still, the sex-differences in this study underscore the importance of a gender perspective on all parts of the MI-journey, including symptom representation and recognition, reasons for delay, diagnosis, treatment and long-term outcome.

There is clear evidence that early treatment, especially within the first "golden hour" results in a considerable survival benefit [25]. There are conflicting data regarding whether there are gender differences in clinical manifestations and delay before seeking treatment for acute cardiac symptoms. However, in Northern Sweden we found no major gender differences in type of symptoms or time between onset and medical presence as defined in the WHO MONICA manual based on the same population as the present study [26].

### Strengths and limitations

The major strengths of our study was the large number of patients studied over such a long period, 23 years, which is longer than most studies. When analysing mortality from MI it is not enough to analyse in-hospital mortality. A considerable proportion of deaths from MI occur outside hospital. We also included deaths before admission, thereby accurately describing the total burden of disease. The validity of our findings is strengthened by the strict and uniform use of the MONICA criteria over the whole period. It should also be noted that this is not a random sample from the population. The Northern Sweden MONICA registry includes all those who suffered an MI in the defined geographical area with no loss to follow-up.

The main limitation and a major drawback of this study is the upper age limit chosen in the WHO MONICA project. When the project was initiated in the early 1980 s, the focus was on what was regarded as premature cardiovascular disease. Therefore, an upper age limit was set at 65 years. Thus, the results cannot be extrapolated to older age groups or to the total population.

Another limitation of the present study is the introduction of troponins (in the 1990 s) as markers of myocardial injury. These markers were not included in the original MONICA criteria from the early eighties for validation and classification of events. This means that MI definitions before and after the year 2000 differ to some extent. It is complicated to handle this situation when all the hospitals in Sweden changed their diagnostic markers and stopped using the older biomarkers that were described in the MONICA project. Our analysis [16] suggests that this may be the reason for the attenuated decline in incidence of MI noted after 2000, but no impact was noted on case-fatality (*i.e*. proportion dead after 28 days). The switch to troponins would not affect the large proportion of subjects dead before hospital, nor is it probable that the effect would be differential according to sex and thus invalidate our findings.

## Conclusions

We conclude that over a 23-year period the age-adjusted survival after a first MI below the age of 65 years is higher among women than among men due to lower risk for women to die before reaching hospital. Survival is similar in those having an MI after 2000. For both men and women survival improved impressively.

## Competing interests

The authors declare that they have no competing interests.

## Authors' contributions

ME designed the study, analysed data and drafted the manuscript. RMI analysed data and drafted the manuscript. JHJ, DL, KZ and UN drafted and revised the manuscript and contributed to the analysis. DL is the principal investigator for the MONICA Registry. All authors have read and approved the final manuscript.

## Pre-publication history

The pre-publication history for this paper can be accessed here:

http://www.biomedcentral.com/1471-2261/11/1/prepub
